# Nanopore detection of DNA molecules in magnesium chloride solutions

**DOI:** 10.1186/1556-276X-8-245

**Published:** 2013-05-20

**Authors:** Yin Zhang, Lei Liu, Jingjie Sha, Zhonghua Ni, Hong Yi, Yunfei Chen

**Affiliations:** 1 Jiangsu Key Laboratory for Design and Fabrication of Micro-Nano Biomedical Instruments, School of Mechanical Engineering, Southeast University, Nanjing 211189, China

**Keywords:** Nanopore, DNA sequencing, Translocation speed

## Abstract

High translocation speed of a DNA strand through a nanopore is a major bottleneck for nanopore detection of DNA molecules. Here, we choose MgCl_2_ electrolyte as salt solution to control DNA mobility. Experimental results demonstrate that the duration time for straight state translocation events in 1 M MgCl_2_ solution is about 1.3 ms which is about three times longer than that for the same DNA in 1 M KCl solution. This is because Mg^2+^ ions can effectively reduce the surface charge density of the negative DNA strands and then lead to the decrease of the DNA electrophoretic speed. It is also found that the Mg^2+^ ions can induce the DNA molecules binding together and reduce the probability of straight DNA translocation events. The nanopore with small diameter can break off the bound DNA strands and increase the occurrence probability of straight DNA translocation events.

## Background

Nanopore sensor, which is derived from the Coulter counter [1], has been utilized for detection and analysis of various single charged molecules [2-9]. Now, it is a widespread concern as a potential candidate to achieve the ‘$1,000 genome’ goal set by the US National Institutes of Health due to its high speed and low cost performance. In a typical nanopore-sensing experiment, ions and biomolecules are driven by an external transmembrane electric field. Biomolecule passage through the nanopore can cause a characteristic temporary blockade in the trans-pore ionic current. Information of the biomolecules such as length, composition, and interactions with other biomolecules can be extracted from the blockade ionic current. In order to get the structural information of a DNA strand at the single base level, a bottleneck to break through is to control the DNA translocation speed through a nanopore. Intuitively, we can change the applied voltage, salt concentration, viscosity, and electrolyte temperature to reduce the translocation speed [10]. The side effect of this method is the reduction of the signal amplitude, which leads to more difficulties in capturing the very weak ionic current change [11]. Another method is to apply a salt gradient on the electrolyte solution across the pore, which can be used not only to prolong the translocation time but also to enhance the capture rate [12]. Recently, some groups tried introducing positive charges into nanopores as molecular ‘brakes’, which is proved to be an effective approach to increase the attractive force between the negative DNA molecule and the positive nanopore inner wall, thus increasing the duration time more than 2 orders of magnitude [13]. The shortcoming of this method is that the residual ionic current during the DNA translocation is insufficient for direct base identification. Aside from an electric field applied along the nanopore axis direction, Tsutsui et al. added a transverse electric field to slow down the translocation speed of DNA across the nanopore [14]. It is reported that adding a transverse field of 10 mV/nm in a gold electrode embedded silicon dioxide channel can make 400-fold decrease in the DNA translocation speed. Similarly, He et al. reported a method to control the DNA translocation speed by gate modulation of the nanopore wall surface charges. It is found that native surface-charge-induced counterions in the electro-osmotic layer substantially enhance advection flow of fluid, which exerts stronger dragging forces on translocating DNA and thereby lowering the DNA translocation speed. Based on this phenomenon, they regulate DNA translocation by modulating the effective wall surface charge density through lateral gate voltages. The DNA translocation speed can be reduced at a rate of about 55 μm/s per 1 mV/nm through this method [15,16]. Yen et al. [17] and Ai et al. [18] reported that applying positive gate voltage could also induce DNA-nanopore electrostatic interaction, which can regulate the DNA translocation speed. Lately, a functionalized soft nanopore composed of a solid-state nanopore and a functionalized soft layer was demonstrated that can not only increase DNA capture rate by counterion concentration polarization occurring at the nanopore mouth but can also decrease DNA translocation speed in the nanopore through electro-osmotic flow [19]. Stolovitzky's group designed a nanopore with a metal-dielectric sandwich structure capable of controlling the DNA translocation process with a single-base accuracy by tuning the trapping electric fields inside the nanopore [20-22]. This design is verified by molecular dynamics (MD) simulations, but there is no device reported so far due to its difficulty in fabrication. Applying an external force in the opposite direction of the electric field force on DNA could control a DNA strand through a nanopore at a very slow speed. It can be achieved using optical tweezer [23] or magnetic tweezer [24] technologies. However, it is hard to extend these methods to sequence DNA in parallel [25], such as employing thousands of nanopores on a chip concurrently [26].

As we know, counterions in solutions can bind to DNA molecules, which may provide a drag force on the DNA and reduce the translocation speed. Dekker's group found that DNA translocation time in LiCl salt solution is longer than that in KCl or NaCl solutions. Through MD simulation, they elucidated that the root of this effect is attributed to the stronger Li^+^ ion binding DNA than that of K^+^ and Na^+^[27]. The DNA electrophoretic mobility depends on its surface charge density and the applied voltage. If we can adjust the DNA surface charge density, it is possible to actively control the DNA translocation through a nanopore. It has been found that Mg^2+^ could reduce electrophoretic mobility of DNA molecule more than Na^+^ at the same concentration without worrying about changing the DNA molecule charge to a positive value [28]. It is also known that Mg^2+^ is regularly used in adhering the DNA to inorganic surfaces, which may also reduce the DNA mobility. Inspired by the process of reducing effective surface charge density of a DNA molecule and that increasing the attractive force between DNA molecule and nanopore inner surface can retard DNA molecule translocation, we employed bivalent salt solution such as MgCl_2_ to observe the DNA translocation event through nanopores. We hope the two kinds of phenomena occur at the same time, thus extending the translocation time further more.

## Methods

**Figure 1:**
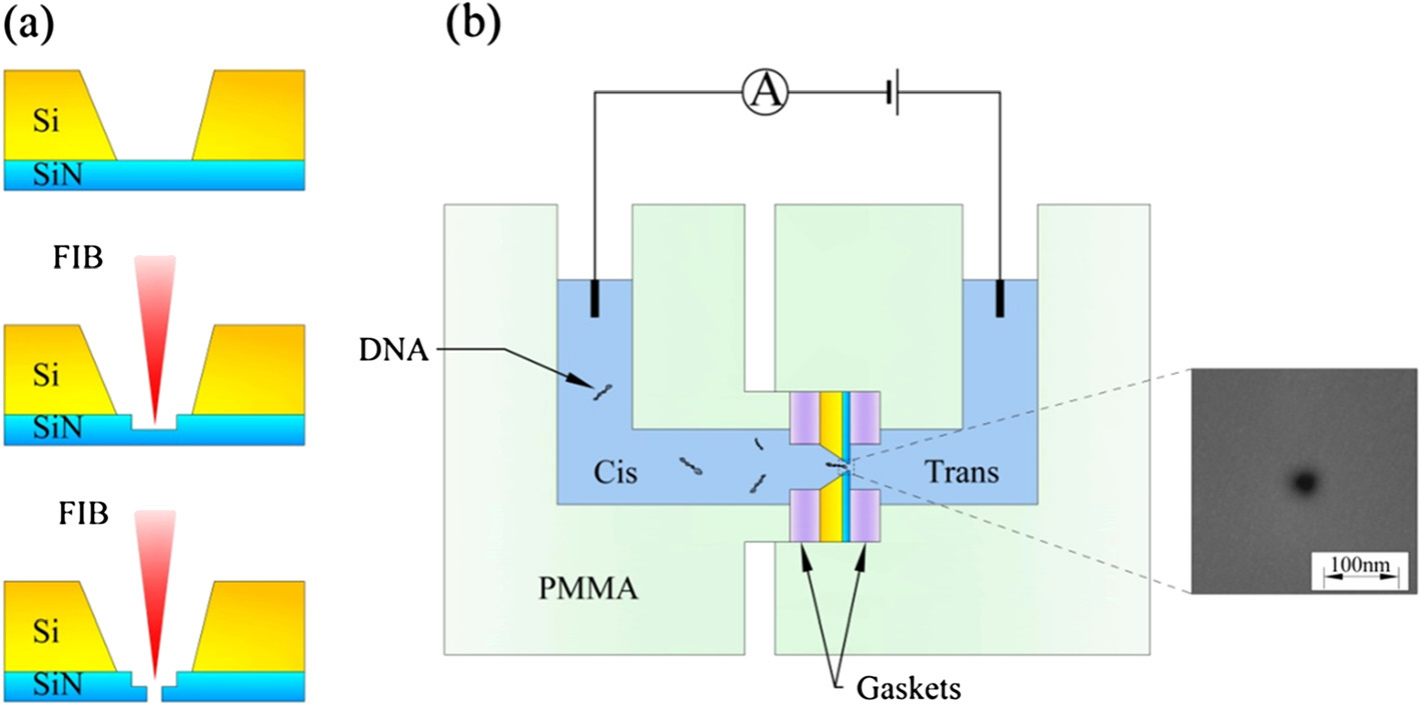
**The setup of measuring the ionic currents through a nanopore.** (**a**) Schematic illustrations of the nanopore fabrication process and (**b**) the microfluidic setup. FIB, focused ion beams; PMMA, polymethylmethacrylate; **Ⓐ**, electrometer.

The fabrication process of a solid-state nanopore is shown in Figure 1a. It starts with the fabrication of a 100-nm thick, low-stress Si_3_N_4_ window (75 × 75 μm^2^) supported by a silicon chip using lithography and wet etching processes. Then, we mill the membrane in a small window with size of 500 × 500 nm^2^ to reduce the membrane thickness to approximately 20 nm. Following the milling process, a nanopore with diameter in several nanometers is drilled on the milled region in the Si_3_N_4_ film. Both the milling and drilling processes are completed by focused ion beams in a dual beam microscope (Helios 600i NanoLab, FEI Company, Hillsboro, USA). The milling process is used to reduce the nanopore length, which can enhance the nanopore sensitivity to biomolecules. Two nanopores are fabricated with diameters of around 7 nm and about 20 nm as shown in the right inset of Figure 1b. The chips with nanopore fabricated on are cleaned in piranha solution and treated in oxygen plasma for 30 s on both sides prior to use. As shown in Figure 1b, the chip is assembled into a polymethylmethacrylate flow cell and sealed by means of silicone elastomer gaskets [29]. Two Ag/AgCl electrodes are immersed in two electrolyte compartments separated by the chip for setting up a transmembrane potential and detecting the transmembrane ionic currents through the nanopore. The ionic current is measured at 100 kHz with low-pass filtering at 10 kHz using a resistive feedback amplifier (EPC10, HEKA Elektronik, Rheinland-Pfalz, Germany). All salt solutions are degassed, filtered, and adjusted to pH 8.0 using 10 mM Tris–HCl and 1 mM EDTA at pH 8.0 at room temperature. The λ-DNA (48.5 kbp, about 16.2-μm long) we used is purchased from Takara Bio, Inc. (Otsu, Japan) and put in the cis chamber (chamber with cathode). A voltage of 600 mV is applied on the trans side. All measurements are taken inside a dark Faraday cage.

## Results and discussion

**Figure 2:**
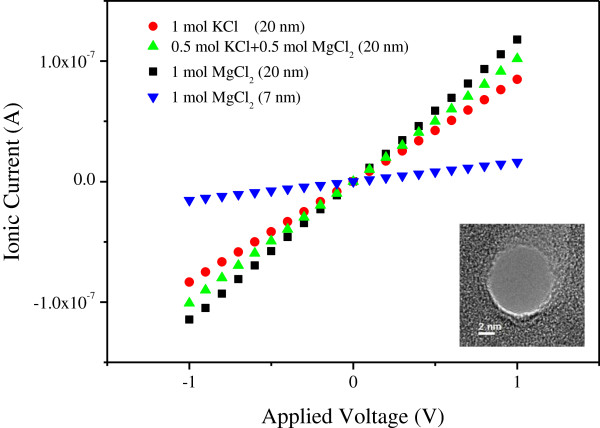
***I*-*V *curves for different nanopores in different solutions.** The inset on the right bottom is a TEM image of a 7-nm diameter nanopore.

Figure 2 shows the current–voltage curves for nanopores with diameters of 7 and 20 nm in various salt solutions. There are four set data representing the open pore ionic conductance, which include three set data for the 20-nm diameter nanopore in 1 M KCl, 0.5 M MgCl_2_ + 0.5 M KCl, and 1 M MgCl_2_ solutions and one set data for the 7-nm diameter nanopore in 1 M MgCl_2_ solution. The open pore ionic conductance of a cylindrical nanopore in high ionic strength solutions with diameter *d*_open_ and thickness *h* can be expressed as [30,31]

(1) $$G=\sigma {\left(\frac{4h}{\pi d_{\text{open}}^{2}}+\frac{1}{d_{\text{open}}}\right)}^{-1},$$

where *σ* is the bulk electrolytic conductivity. In this paper, it is set as *σ*_KCI_ = 9.83 *Sm*^−1^, $\sigma_{{\mathrm{MgCl}}_{2}}=12.3\;Sm^{-1}$ at 18°C for 1 M KCl and 1 M MgCl_2_ according to reference [32]. Given the bulk electrolytic conductivity, the open pore conductance for a nanopore can also be estimated from formula (1). Based on formula (1), it is estimated that the open pore conductance for the 20-nm diameter nanopore in the three type solutions of 1 M KCl, 0.5 M MgCl_2_ + 0.5 M KCl, and 1 M MgCl_2_ should depend directly on the bulk electrolytic conductivity and the salt concentration. The predicted ratio for the open pore conductance in the above three solutions is 1:1.13:1.25, which agrees well with the measured value of 1:1.19:1.37 extracted from Figure 2. The open pore conductance for the 7-nm diameter nanopore can also be calculated. The predicted result is 18.56 nS, which is consistent with the experimental results, too.

**Figure 3:**
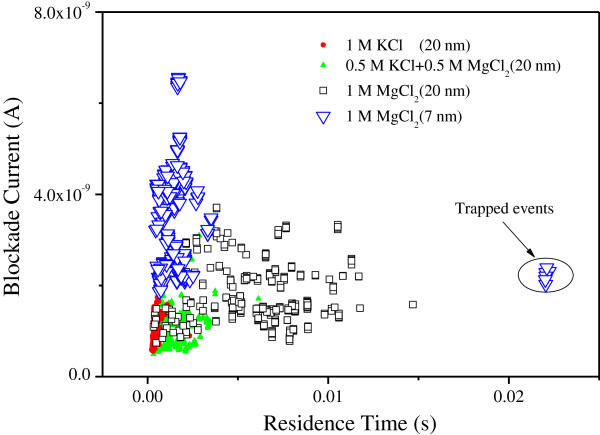
Scatter plot of the event residence time versus its blocked ionic current amplitude.

Figure 3 presents the scatter plots of the event residence time *t*_d_ versus blockade current amplitude ∆*I* for different experiment conditions. Once a DNA strand enters the nanopore, it will block the ions in and out the nanopore and cause ionic current reduction. The amplitude of the blocked ionic current can be expressed with Kowalczyk's model [31],

(2) $$\begin{array}{l} \mathrm{\Delta I}=V\cdot \mathrm{\Delta G}=V\cdot \left(G_{\text{open}\;\text{pore}}-G_{\text{with}\;\mathrm{DNA}}\right)\\ \;=V\cdot \left(\sigma {\left(\frac{4h}{\pi d_{\text{open}}^{2}}+\frac{1}{d_{\text{open}}}\right)}^{-1}-\sigma {\left(\frac{4h}{\pi d_{\text{with}\;\mathrm{DNA}}^{2}}+\frac{1}{d_{\text{with}\;\mathrm{DNA}}}\right)}^{-1}\right), \end{array}$$

where *V* is the applied bias voltage, and $d_{\text{with}\;\mathrm{DNA}}=\sqrt{d_{\text{open}}^{2}-d_{\mathrm{DNA}}{}^{2}}$ is the effective diameter of the nanopore with DNA in the pore. According to formula (2), the blocked ionic current amplitude (∆*I*) is linearly proportional to *σ* for the nanopore with the same diameter. Therefore, the amplitude of the blockade ionic current for DNA translocation through the nanopore in MgCl_2_ solution is expected to be larger than that in the KCl solution with the same molar concentration because the former has a high electrolytic conductivity. Unfortunately, the results as shown in Figure 3 do not meet such prediction. The 20-nm diameter nanopore produced a little difference in the amplitude of the blocked ionic current in the three salt solutions (1 M KCl, 0.5 M KCl + 0.5 M MgCl_2_, and 1 M MgCl_2_). As shown in Figure 3, the red solid circle points denote the events for the 48.5 kbp λ-DNA translocation through the nanopore in 1 M KCl solution. The green solid triangle points stand for the events that occurred in 0.5 M KCl + 0.5 M MgCl_2_ solution, and the black open rectangle symbols stand for the events in 1 M MgCl_2_ solution. The three symbols almost overlap with the black open rectangle symbols which are located a little higher. This result tells us that the electrolyte conductivity is only one of the factors that affect the blockade ionic currents.

In Figure 3, some outliers we call as ‘trapped events’ have been observed in 1 M MgCl_2_ experiments. Although the probability is small, the duration time of these events is 22 ms, about 17 times of the other events in 1 M MgCl_2_ experiments. As we know, Si_3_N_4_ surface in aqueous solution at pH 8.0 is negatively charged. The correlations between Mg^2+^ ions on both the negatively charged DNA and the Si_3_N_4_ surface can generate a net attraction force and then help stick the DNA into the nanopore, but the phenomenon only obviously occurred for the 7-nm diameter nanopore experiments. This is because the gap between the DNA and the inner surface of the nanopore is also increased with the increasing nanopore diameter. With the increase of the gap, the net attraction force is not strong enough to stick the DNA, which leads to the trapped events unremarkable in the 22-nm diameter nanopore.

**Figure 4:**
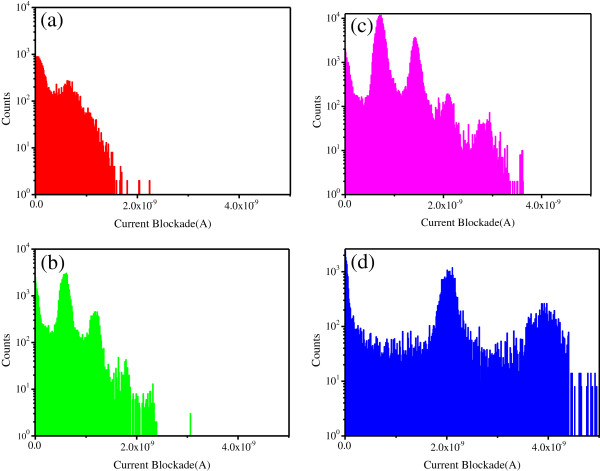
**Current blockade histograms in different experiment conditions.** (**a**) In 1 M KCl solution for the 20-nm diameter nanopore, (**b**) in the mixed solution with 0.5 M KCl + 0.5 M MgCl_2_ for the 20-nm diameter nanopore, (**c**) in 1 M MgCl_2_ solution for the 20-nm diameter nanopore, and (**d**) in 1 M MgCl_2_ solution for a 7-nm diameter nanopore.

From Figure 3, we find not only the blockade current amplitude and duration time but also the event point dispersion degree increase with the increasing Mg^2+^ ion concentration. In order to further analyze the translocation events, we plot the current blockade histograms on a semi-logarithmic scale in Figure 4. There are some peaks in each histogram of the current data, and they correspond to different translocation events. We can define a variable *N* to describe the DNA spatial state, the value of which represents the number of base pairs in the cross-section perpendicular to the pore axis. The lowest blockade current value peak is interpreted as a single DNA molecule in the nanopore in a linear configuration [3]. We call such event with *N* = 1 as ‘event A’. The other peaks correspond to the events of folded DNA molecule translocation or several parallel straight DNA in the pore, or both. We call those events with *N* > 1 as ‘event B’. There is only one obvious peak in Figure 4a, and some other discrete points, which is much larger than the first peak of the blockade current value. This is interpreted as event A occurs with high frequency in KCl experiments. However, due to the relatively large diameter (approximately 20 nm), several DNA strands are also able to thread the nanopore simultaneously or a DNA strand could translocate in a folded state [33], which may cause a higher blocked ionic current as shown those as discrete points in Figure 4a. When DNA molecules pass the same nanopore in MgCl_2_ solutions, it is reflected that there are four peaks in Figure 4b and even five peaks in Figure 4c. This indicates that event B is easy to happen in MgCl_2_ solution. With increasing Mg^2+^ concentration, this phenomenon becomes more obvious. Comparing the occurrence number of event B in Figure 4a,b,c, it is concluded that Mg^2+^ ions play dominant role in inducing several DNA strands binding together or a single DNA strand being folded. In a monovalent salt solution, as shown in Figure 4a, the attraction force between neighboring DNA strands is weak and the event B is seldom observed. However, in the divalent MgCl_2_ solutions, event B occurred with a larger number and several peaks appeared obviously in Figure 4b,c. This is attributed to the presence of the Mg^2+^ ions, which induces the attraction force between the neighboring DNA strands. Similar phenomenon is also reported in reference [34]. With the increase of the Mg^2+^ ion concentrations, the attraction force becomes strong enough that it can make the formation of minor-grove-to-minor-grove bound state for DNA molecules bridged by Mg^2+^ ions. In the 1 M MgCl_2_ electrolyte, thermal fluctuations can only transitorily increase the inter-DNA distance but cannot break the bound state [34]. So, event B with *N* = 4 is more often observed in Figure 4c. This implies that more DNA strands can be bound together or a single DNA strand is folded with many sections induced by the high concentration of Mg^2+^ ions. However, the bound state can be broken off by reducing the nanopore diameter. As shown in Figure 4d, the number of peaks is reduced to two for the DNA passing through a 7-nm diameter nanopore in the 1 M MgCl_2_ solution. With the decrease of the nanopore diameter, the bounded or folded DNA with more than two sections (*N* > 2) has to be broken off or pulled straight to thread through the small aperture.

**Figure 5:**
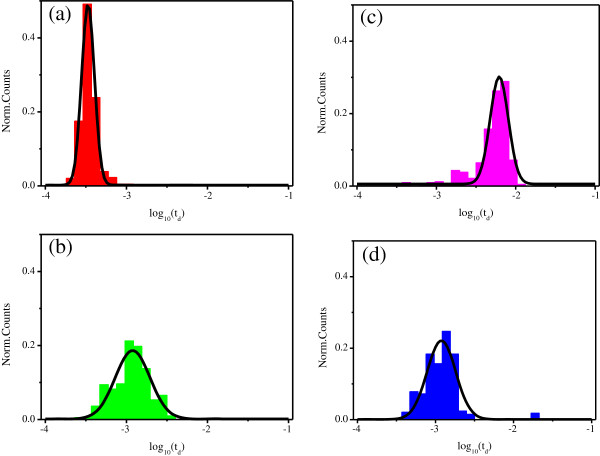
**The duration time histograms in a logarithmic scale.** (**a**) In 1 M KCl solution for the 20-nm diameter nanopore, (**b**) in the mixed solution with 0.5 M KCl + 0.5 M MgCl_2_ for the 20-nm diameter nanopore, (**c**) in 1 M MgCl_2_ solution for the 20-nm diameter nanopore, and (**d**) in 1 M MgCl_2_ solution for a 7-nm diameter nanopore.

Figure 5 displays the duration time histograms in a logarithmic scale. Solid curves are the Gaussian fit to the histogram. Figure 5a shows the residence time peak at 0.36 ms, but Figures 5b,c respectively show peaks in 1.21 and 6.19 ms for the same diameter nanopore. The duration time increases with the increase of the Mg^2+^ ion concentration. As we know, the net charge of a DNA molecule sensitively depends on the valence of counter ions [35]. K^+^ and Mg^2+^ ions all could reside in the negatively charged pockets formed by phosphate groups of the DNA backbone. However, Mg^2+^ ions bond stronger and last longer than K^+^ ions. Therefore, the net charge of DNA molecules in MgCl_2_ electrolyte is lower than that in KCl electrolyte. With the decrease of the surface charge density in DNA strands, the DNA electrophoretic mobility will be reduced under the action of the same external applied voltage, thus increasing the translocation time. Comparing the translocation time between Figure 5c,d, it is found that the translocation time for DNA strand through the 7-nm diameter nanopore in 1 M MgCl_2_ solution is about 1.19 ms, much shorter than the duration time of 6.19 ms for the DNA strand through the 20-nm diameter nanopore in the same solution. The only difference between the two cases is the nanopore diameter. It is reasonable that event B is the main cause of the longer average duration time, as shown in Figure 5c. Event B refers to several types of DNA spatial states in translocating a nanopore. One type is a single strand DNA translocating through a nanopore in more than two folded states. In this case, the length of the two-folded or more than two-folded DNA should be shorter than its straight state, and it will cost shorter time to translocate through the nanopore. Event B also includes several DNA strands binding together to pass through the nanopore. When the bounded DNA strand passes through the 20-nm diameter nanopore, the drag force on the DNA strand coming from the nanopore will be strong and extends the duration time. It is easier for several bounded DNA strands to pass through the 20-nm diameter nanopore than through the 7-nm diameter nanopore; this will extend the averaged duration time for the 20-nm diameter nanopore.

**Figure 6:**
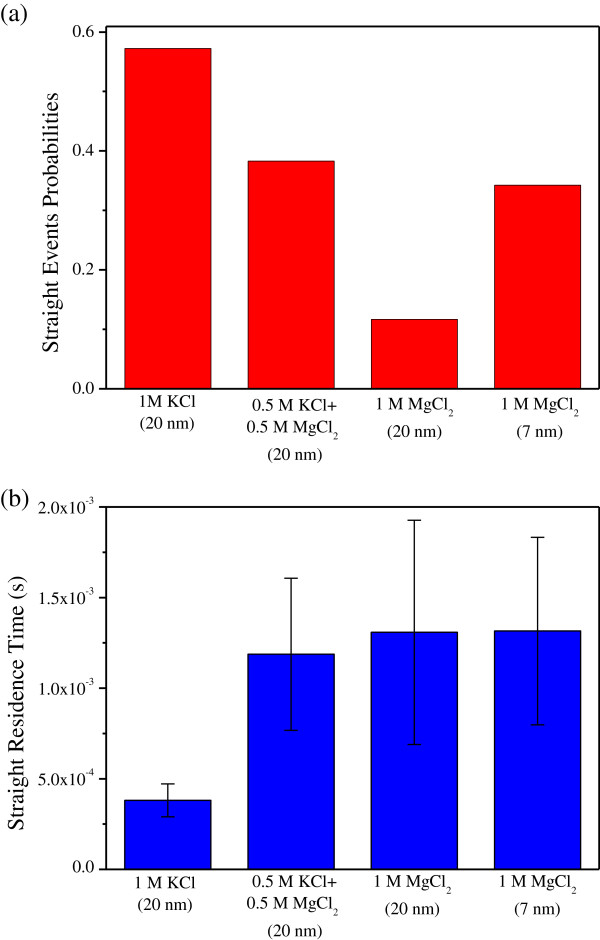
**Straight state translocation events.** (**a**) Probabilities in different experiment conditions. (**b**) Average residence times in different experiment conditions.

In fact, we are more interested in the average translocation time for event A. So, we distinguish event A from B, and then give the happening probability and the average duration time of event A. As shown in Figure 6a, for the 20-nm diameter nanopore, the probability of straight translocation events falls sharply in an electrolyte rich in Mg^2+^ ions. This phenomenon is consistent with our analysis, but it is disadvantage for DNA detection and analysis. However, aperture reduction can raise the probability of DNA molecule straight translocation event from 11.7% to 34.3%, which may ease this problem. From Figure 6b, we can see for the 20-nm diameter nanopore that event A averaged duration time also rises with the increase of Mg^2+^ ion concentration, as we expected. It is 1.31 ms for 1 M MgCl_2_ solution, about three times longer than that for the same DNA in 1 M KCl solution. We also found that the translocation time for the 7-nm diameter nanopore is 1.32 ms, almost the same as that for the 20-nm diameter nanopore. So, we can conclude that the translocation time of event A does not depend so much on the diameter of a nanopore.

## Conclusion

In summary, the duration time for DNA translocation through a nanopore can be extended with the use of MgCl_2_ electrolyte. The side effect is that Mg^2+^ ions may induce more DNA strands binding together, which is harmful to do DNA sequencing in MgCl_2_ electrolyte. Reducing the nanopore diameter can effectively reduce the occurrence number of the folded DNA translocation events. So, we can say that theMgCl_2_ solution is a good choice for nanopore DNA sequencing experiments if nanopore diameter can be reduced further.

## Competing interest

The authors declare that they have no competing interests.

## Authors’ contributions

YZ and YC designed the experiments. YZ carried out the total experiment and participated in the statistical analysis. ZN, HY, and YC guided the experiment. YZ, LL, JS, ZN, and YC discussed the results and co-wrote the manuscript. All authors read and approved the final manuscript.

## Authors’ information

YZ is a PhD candidate of Mechanical Design and Theory at the School of Mechanical Engineering, Southeast University, Nanjing, P.R. China. He is interested in nanopore fabrication and nanopore biosensing. LL is an assistant professor of Mechanical Design and Theory at the School of Mechanical Engineering, Southeast University, Nanjing, P.R. China. His research interests are biomolecule sensing and biodegradable materials design. JS is an assistant professor of Mechanical Design and Theory at the School of Mechanical Engineering, Southeast University, Nanjing, P.R. China. Her research interest is micro-nano fluidic device design. ZN is a professor of Mechanical Manufacture and Automation at the School of Mechanical Engineering, Southeast University, Nanjing, P.R. China. His research interests are minimally invasive medical devices and microfluidic diagnostic device design and manufacture. HY is a professor of Mechanical Manufacture and Automation at the School of Mechanical Engineering, Southeast University, Nanjing, P.R. China. His research interest is advanced manufacturing technology. YC is a professor of Mechanical Design and Theory at the School of Mechanical Engineering, Southeast University, Nanjing, P.R. China. His research interests cover heat transfer, tribology, micro-nano fluidics, and micro-nano biomedical instrument.
